# Key role of UBQLN2 in pathogenesis of amyotrophic lateral sclerosis and frontotemporal dementia

**DOI:** 10.1186/s40478-019-0758-7

**Published:** 2019-07-18

**Authors:** Laurence Renaud, Vincent Picher-Martel, Philippe Codron, Jean-Pierre Julien

**Affiliations:** 1 Department of Psychiatry and Neuroscience, Laval University, Quebec, Canada; 2 CERVO Brain Research Center, 2601 Chemin de la Canardière, Québec, QC G1J 2G3 Canada; 3 UMR CNRS 6015, INSERM U1083, University of Angers, Angers, France

**Keywords:** Amyotrophic lateral sclerosis (ALS), Ubiquilin-2 (UBQLN2), TAR DNA-binding protein 43 (TDP-43), Ubiquitin-proteasome system (UPS), Autophagy, Animal models

## Abstract

Ubiquilin-2 (UBQLN2) is a member of the ubiquilin family, actively implicated in the degradation of misfolded and redundant proteins through the ubiquitin-proteasome system and macroautophagy. UBQLN2 received much attention after the discovery of gene mutations in amyotrophic lateral sclerosis and frontotemporal dementia (ALS/FTD). The abnormal presence of positive UBQLN2 inclusion in the cytosol of degenerating motor neurons of familial and sporadic forms of ALS patients has been newly related to neurodegeneration. Only recently, data have emerged on its role in liquid-liquid phase separation, in stress granule development and in the formation of secondary amyloid structures. Furthermore, several animal models are available to investigate its involvement in TDP-43 pathology and neuroinflammation in ALS. This review addresses the molecular pathogenetic pathways involving UBQLN2 abnormalities which are converging toward defects in clearance mechanisms. UBQLN2.

## Introduction

Amyotrophic lateral sclerosis (ALS) is a progressive and fatal disorder associated with degeneration of upper and lower motor neurons [55]. The disease is characterized by progressive and diffuse paralysis leading to death from respiratory failure within 2 to 5 years of symptoms onset. Approximately 90% of ALS cases are sporadic (sALS), occurring without any familial history of the disease. The remaining 5–10% of cases constitute the familial form of ALS (fALS). Several genes have been implicated in fALS, notably the hexanucleotide repeat expansion in *C9ORF72* (Chromosome 9 open reading frame 72), *SOD1* (Superoxide dismutase 1), *TDP-43* (TAR DNA-binding protein 43) and *FUS* (Fused in Sarcoma) [28, 53, 61]. In 2011, Deng et al. first identified missense mutations in the Ubiquilin-2 (UBQLN2) gene in large ALS and ALS/FTD families, which has placed this protein as a new central actor in the physiopathology of ALS.

UBQLN2 is a shuttle protein implicated in the ubiquitin-proteasome system (UPS). One of the most actively studied mechanism in UBQLN2-related pathology is the mutant-related dysfunction of the UPS. However, its implication in the cytoplasmic mislocalization of TDP-43 into insoluble aggregates is also well described in ALS. More recently, ALS-linked mutations in *UBQLN2* gene were found to be associated with dysfunction of autophagy [24], neuroinflammation [49, 50] and formation of stress granules (SGs) [9]. These recent data surely placed UBQLN2 as an essential player in noxious protein accumulation and clearance pathways in ALS and FTD pathogenesis.

In this review we will describe the structure and the function of UBQLN2, and we will propose an integrative mechanism for the pathogenesis of UBQLN2 in ALS and FTD.

## UBQLN2

### Structure

**Fig. 1:**
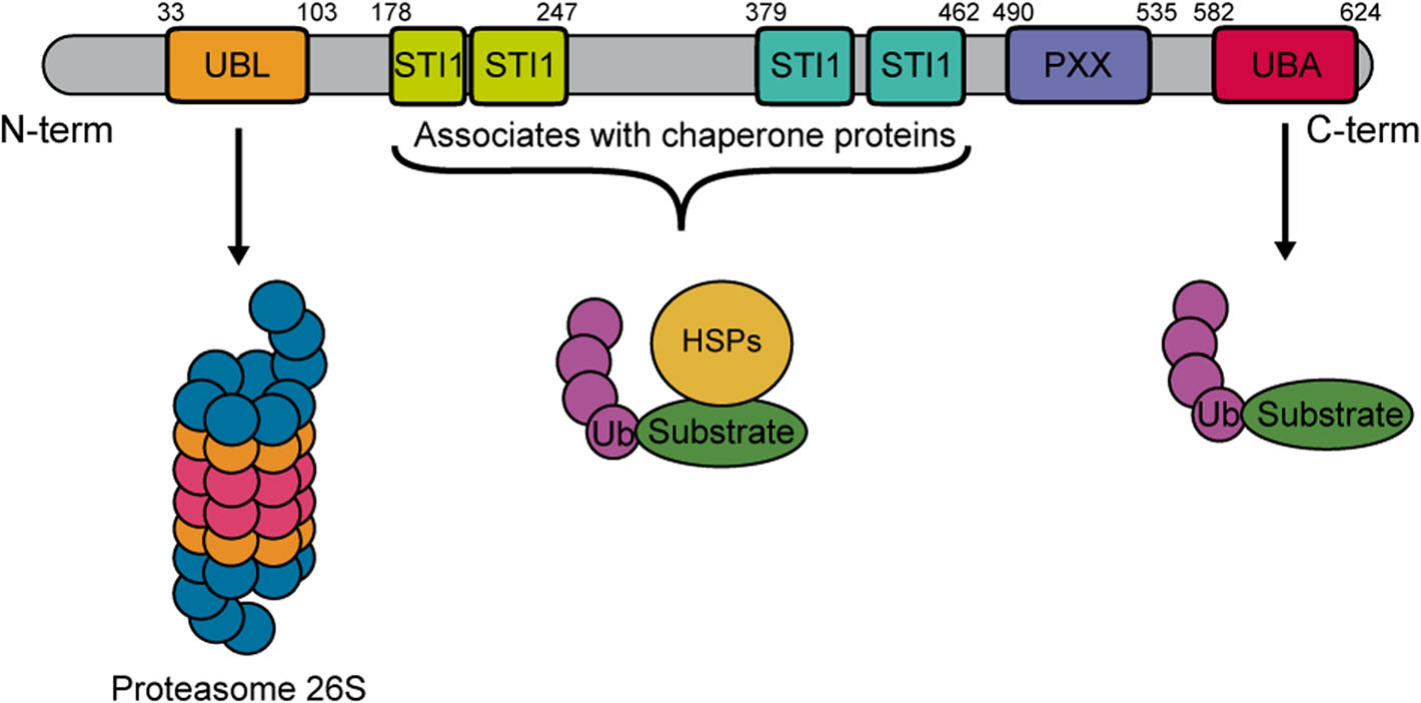
Schematic representation of the domain architecture of the human *Ubiquilin-2* gene. *UBQLN2* gene is in the chromosome Xp11.21 and have only one coding exon. UBQLN2 possess a ubiquitin-like domain (UBL) domain on the N-terminal that interacts with the proteasome and a ubiquitin-associated domain (UBA) on the C-terminal required for the UPS activity. *UBQLN2* gene also arbors four stress-induced protein 1 (STI-1)-like motif and a proline-rich repeat domain containing 12 PXX repeats. UBL: Ubiquitin-like domain; STI-1: Stress-induced protein 1; UBA: Ubiquitin-associated domain; HSP70: Heat-shock protein 70; Ub: Ubiquitin

The *UBQLN2* gene, also called *Chap1*/*Dsk2* or *PLIC*, is located on the Xp11.21 chromosome. UBQLN2 is a single-exon coded, 66 kDa protein member of the ubiquilin family [12, 29]. UBQLN2 has a ubiquitin-like domain (UBL) in its N-terminal that can interact with the proteasome, and a ubiquitin-associated domain (UBA) on the C-terminal which recognizes the polyubiquitin chains on marked proteins [63]. UBQLN2 contains four stress-induced protein 1-like domains (STI-1 like), located between the residues 178–247 and 379–462, which are involved in the interaction with heat-shock proteins and autophagy mediators. UBQLN2 has also a unique PXX domain containing 12 tandem repeats implicated in protein-protein interactions [1, 12] (Fig. 1). This domain differentiates UBQLN2 from other members of the ubiquilin family. Interestingly, most of the mutations identified in the *UBQLN2* gene are located in the PXX domain. Yet, it is unclear how the mutations impact the secondary structure and functions of the PXX domain. The 3-dimensional structure of UBQLN2 is formed by five β-strands, an α-helix of 3.5 turns and an additional 3_10_-helix [63].

### Biological functions

#### UBQLN2 and the ubiquitin-proteasome system

**Fig. 2:**
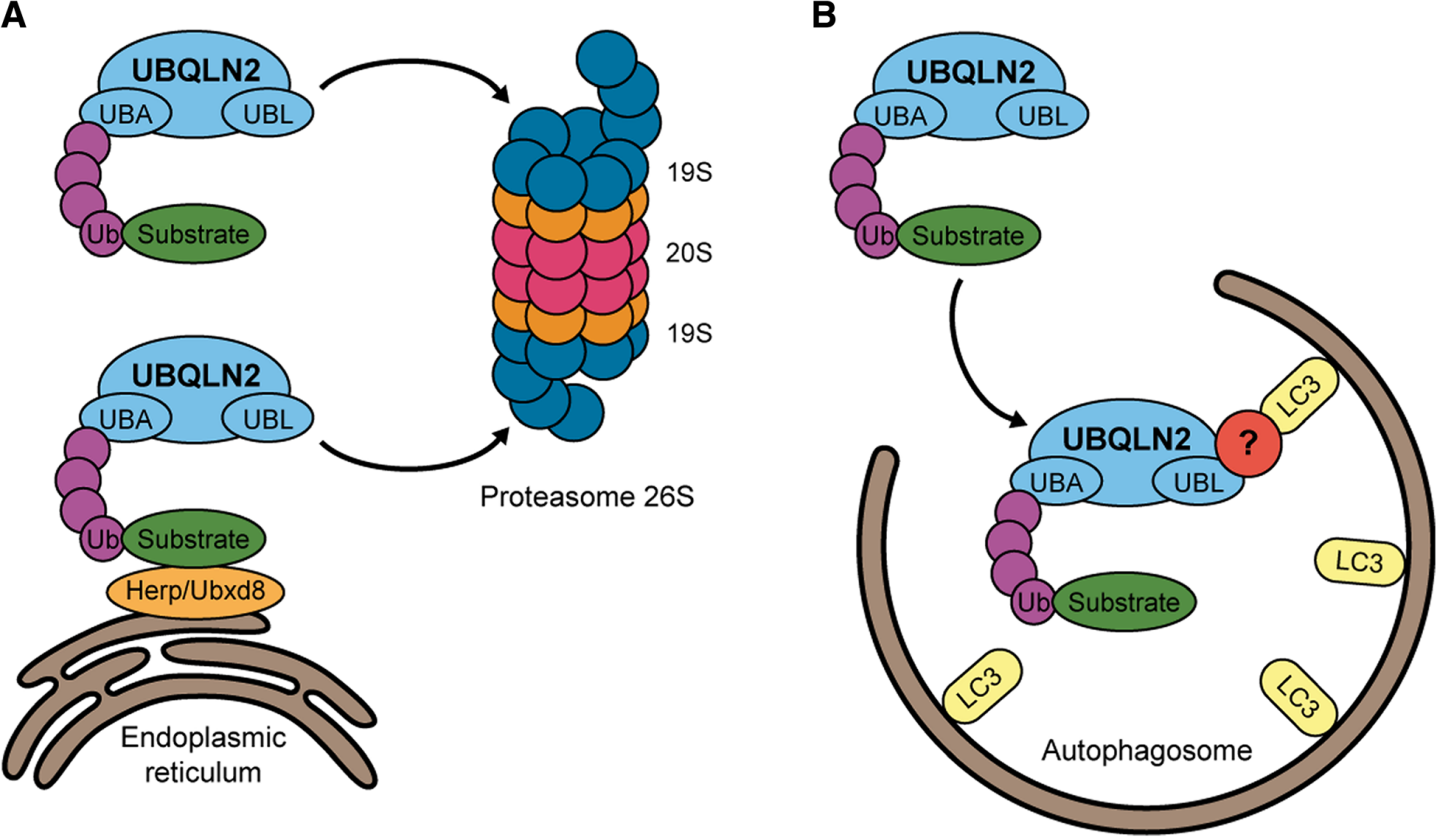
Roles of UBQLN2 in protein degradation in both the UPS and the autophagy systems. **a** UBQLN2 targets ubiquitinated substrates and interacts with ER proteins involved in endoplasmic-reticulum-associated protein degradation (ERAD), such as Herp and Ubxd8, to drive substrates degradation by the 26S proteasome. **b** UBQLN2 is also implicated in the macroautophagy and interacts with LC3 indirectly through an unknown mechanism and can deliver ubiquitinated proteins bound to the ubiquitin-associated domain (UBA). UBQLN2: Ubiquilin-2; Ub: Ubiquitin; LC3: Microtubule-associated protein 1 light chain 3, Herp: Homocysteine-induced endoplasmic reticulum protein, isoform A. Ubxd8: ubiquitin regulatory X domain-containing protein 8

UBQLN2 is located in the cytosol and has been reported to be mainly expressed in the brain, spleen, heart, liver, pancreas and other tissues [69]. The other members of the ubiquilin family have different tissue expression. UBQLN1 is expressed ubiquitously whereas UBQLN3 is expressed only in testis. UBQLN4 has the same expression profile than UBQLN2. UBQLN2 is mainly involved in protein homeostasis, directing misfolded or redundant proteins towards the proteasome. Proteins marked by ubiquitin, a highly conserved 76-residue polypeptide, are destined to get degraded in a three-step enzymatic cascade [63]. First, the ubiquitin activating enzyme E1 is binding ubiquitin to the protein by building a high-energy thiol ester intermediate. Then, the activated ubiquitin is shuttled from E1 to E2, building another high-energy thiol ester intermediate. A third-class enzyme, the ubiquitin ligase E3, mediates the covalent conjugation of polyubiquitin chains to a lysine residue of the specific UPS substrates. Polyubiquitinated proteins are then recognized by the UBL domain of UBQLN2 and brought to the S5a proteasomal cap of the 26S proteasome for degradation [39] (Fig. 2a). Likewise, UBQLN2 can be activated by heat shock protein 70 (HSP70) [24]. UBQLN2 is inactive under resting conditions but it becomes activated when HSP70 binds to proteins, generating an exposure of a UBQLN2-binding site. The activation of UBQLN2 promotes its binding to the 26S proteasome to form a degradation-competent complex.

Furthermore, UBQLN2 has been implicated in the degradation of endoplasmic reticulum (ER) proteins. UBQLN2 can interact with the ubiquitin regulatory X domain-containing protein 8 protein (Ubxd8), which is localized in the ER membrane and it facilitates the transport of endoplasmic-reticulum-associated protein degradation (ERAD) substrates to the cytosol [66]. UBQLN2 can also interact with Herp (homocysteine-induced ER protein) an ER membrane protein activated in stress conditions which induce the cellular protection by stabilizing ER Ca^2+^ homeostasis and maintaining mitochondrial function (Fig. 2a) [32].

#### UBQLN2 and autophagy

The implication of UBQLN2 in autophagy is now well established. Autophagy, specifically macroautophagy, is crucial for the bulk turnover of redundant cellular components, including protein aggregates and damaged organelles. A specific region of the cytosol is isolated in an intracellular double membrane stretch called autophagosome, secondarily fusing with lysosomes for degradation [23, 42, 43, 68]. UBQLN2 can indirectly interact with the membrane protein LC3, a marker for starvation-induced autophagy, of autophagosomes through its UBA domain to promote autophagosome formation and lysosomal fusion (Fig. 2b) [42, 54]. Interestingly, UBQLN2 was found to be recruited to Optineurin (OPTN)-containing vesicles in Neuro2a and HeLa cells co-transfected with wild-type myc-UBQLN2 and wild-type or E50K HA-OPTN [45]. Those vesicles are positives for autophagy markers, such as p62, LC3, autophagy-related protein 9a (APG9A) and autophagy-related 16 (ATG16). Likewise, a study with ChAttTA-9 transgenic rats crossed with TRE-UBQLN2^P497H^ measured the autophagic flux with an LC3 turnover assay [7]. LC3-II is one of the isoforms of LC3 and is broadly used to measure autophagic flux since its reduced levels is related to impaired autophagy. Transgenic rats used in the study showed significant decreased levels of LC3-II from 6 months of age. The same result was observed for ATG7, another autophagy component essential for autophagosome formation [7]. Thus, those results suggested that mutant UBQLN2^P497H^ compromised autophagy-lysosomal pathways in an age-related manner.

## Ubiquilin-2 in amyotrophic lateral sclerosis

### UBQLN2 mutations in familial ALS

*UBQLN2* mutations were first identified in five unrelated families, suffering of ALS/FTD [12]. From this analysis, five mutations were found in the PXX domain and all consisted of substituted proline residues (P497H, P497S, P506T, P509S and P525S). Subsequently, more than 10 other mutations have been reported [11, 13, 16, 18, 20, 26, 41, 46, 59, 60, 64] (Table 1). Albeit most of mutations have been detected in the PXX domain, some of them were also found in the STI1 domains or between domains [62]. The authors examined 47 post-mortem spinal cord samples of a wide variety of ALS cases, such as sALS (*n* = 23), fALS without mutations in SOD1, TDP-43 and FUS (*n* = 5), ALS with dementia (*n* = 5) and fALS with G298S mutation in TDP-43 (*n* = 21) [12]. Also, brain autopsy samples of cases harboring UBQLN2^P506T^ mutation were analysed. All fALS cases exhibited co-localization of UBQLN2 and TDP-43 cytoplasmic inclusions in the brain and the spinal cord neurons [12]. Interestingly, UBQLN2 was also found to be colocalizing with TDP-43 in the spinal cord of sALS patients, making it a component of the neuronal inclusions in patients affected with the sporadic form of the disease, suggesting a central role for this protein in ALS pathology [12, 17].

**Table 1:** UBQLN2 mutations in ALS patients

Forms of ALS	Age at onset (years)	Mutations	Domain	Clinical features	Neuropathology	Ref.
Familial	16 to 71	P497H P497S P506T P509S P525S	PXX	Earlier onset in male (33.9 ± 14.0) than female (47.3 ± 10.8), ALS, ALS/FTD and pure FTD	TDP-43/UBQLN2 NCI in spinal cord and hippocampus	[12]
Familial	30 to 76	T487I	PXX	Earlier onset in male (39.5 ± 10.4) than female (51.2 ± 14.3), pure ALS, bulbar and spinal onset	FUS/UBQLN2/TDP-43/ubiquitin NCI in the spinal cord	[64]
Sporadic	51 to 73	Q425R A282V A283T	Outside domains	ALS and pure FTD, spinal onset	n/a	[59]
Familial	30 to 57	P506S P533L M446R V538 L N439I	PXX	ALS and ALS/FTD, spinal and bulbar onset	n/a	[18]
Sporadic	46 to 59	S155 N P189T	S155 N: outside P189T: STI-1	Pure ALS, bulbar and spinal onset	n/a	[11]
Familial	4 to 63	P497L	PXX	Phenotype diversity: Choreoathetoid movements, dysarthria, spastic paralysis, ALS, FTD	UBQLN2/TDP-43 NCI in brain stem and hippocampus, striatal atrophy, cerebral atrophy	[16]
Familial and sporadic^a^	27–62	P494L P500S P506A A488T	PXX A488T: outside	ALS and spastic paraplegia	n/a	[60]
Sporadic	62	M392 V	STI1	Pure ALS	n/a	[26]
Sporadic	14–16	M392I	STI1	Madras-type MND	n/a	[46]
Familial	52	p.Gly502_Ile504del	PXX	Bulbar ALS	n/a	[41]
Sporadic	30–77	S346C S400G P440L	Outside STI1	Pure FTD (S346C) and pure ALS	n/a	[13]

*n/a* not applicable, *NCI* neuronal cytoplasmic inclusions

^a^ A488T was found in a sporadic case

### Proteasome and autophagy impairment

The degradation of misfolded or redundant proteins is critical for the maintenance of cellular health, specifically in neurons which are not proliferating cells. There is evidence that UBQLN2 mutations can provoke proteasome as well as autophagy impairments. First, an accumulation of the proteasome efficacy reporter Ubiquitin^G76V_^GFP was detected in Neuro2a cells expressing mutant UBQLN2^P497H^ [12]. They further analyzed the dynamic of this reporter after blocking new protein synthesis with cycloheximide for 0, 2, 4 and 6 h in cells. The rates of Ubiquitin^G76V_^GFP degradation were significantly slower in UBQLN2 mutations (P497H and P506T) when compared to the WT, suggesting the impairment of the protein degradation pathway by UBQLN2 mutations. Another group took advantage of the endogenous Myc as a proteasome efficiency reporter, as it is quickly degraded by the proteasome when its synthesis is blocked by cycloheximide [6]. In HeLa cells expressing mutant UBQLN2 (P497H, P497S, P506T, P509S or P525S) and treated with cycloheximide, a delay in the degradation of Myc was observed as compared to untransfected cells. However, when a chymotrypsin-like test was used to measure efficiency of the proteasome, no dysfunction of the proteasome was detected, which is contradictory with the previous study. Other studies suggested that mutant IVm-UBQLN2 could impede the UPS by reducing the delivery of ubiquitinated proteins to the proteasome [6, 44]. Indeed, ALS-linked mutation in UBQLN2 led to accumulation of ubiquitinated high-molecular-weight complexes (HMWCs). Deletion of the UBL domain of mutant UBQLN2 sequestered ubiquitinated substrates from both the UPS and autophagy (mATG9 and ATG16L1), resulting in an enhanced HMWCs accumulation [44]. Those results suggest that mutant UBQLN2 potentially interferes with the degradation of the polyubiquitinated substrates. Likewise, deletion of UBQLN2 UBL domain impaired the clearance of unfolded nascent polypeptide chains while UBQLN2 levels remained unaffected [24]. Recently, our group highlighted that overexpression of mutant UBQLN2^P497H^ in Neuro2A cells sequestered lys^48_^ bound ubiquitin and resulted in a reduction in the efficiency of the proteasome [50]. This also supports the evidences that mutant UBQLN2 is actively impairing the UPS pathway.

Another study reported, with a knocked-in hUBQLN2^P506T^ mouse, that UBQLN2 works with the HSP70 system for proteasomal degradation of insoluble ubiquitylated protein aggregates [24]. Under non-stressed conditions nor mutations, UBQLN2 is inactive in homo- or heterodimers. In presence of HSP70 clients, UBQLN2 binds itself to HSP70 and associated ubiquitinated aggregated/misfolded proteins. HSP70-HSP110-dependent disaggregase activities can pull aggregated proteins apart, allowing UBQLN2 to form a HSP70-client-UBQLN2-proteasome degradation complex. UBQLN2 can then act as a proteasomal shuttle by connecting ubiquitylated proteins to the proteasome for degradation, leading to the proteolysis of the client. However, mutant UBQLN2 no longer recognized client-bound HSP70 and remained in its inactive phase, leading to accumulation of misfolded/aggregated proteins [24]. Defect in HSP70 binding was later confirmed in ALS patients’ lymphoblasts with P494L, P497H or P506A mutations in UBQLN2 [60]. They also confirmed the role of UBQLN2 as a shuttle factor for HSP70-bound substrates which was previously shown [24]. Thus, activation of UBQLN2 in order to bind to HSP70 seems to represent an important step to accomplish its normal degradation function.

Lastly, optineurin (OPTN) is a ubiquitin-binding multifunctional adaptor protein and is mainly implicated in the autophagy processes. It has also been proposed to be an important factor implicated in regulation of inflammation [38]. UBQLN2 and OPTN can be localized to the same cellular compartment, the Rab11-positive endosomal vesicles [45]. This observation led to propose that these proteins are involved in the same pathological processes in motor neurons diseases. Indeed, OPTN+/UBQLN2+ vesicles showed features of recycling endosomes and were positives for the key autophagy-regulating molecules p62, LC3, mAPG9L1, ATG16, and the autophagy initiator ULK1, suggesting that these vesicles are involved in protein homeostasis [45]. Thus, OPTN and UBQLN2 are implicated in protein homeostasis of the endosomal system via autophagy. Disrupting the function of these vesicles may contribute to development of ALS.

### Direct interaction with TDP-43 promoting aggregation

It has been reported that cytoplasmic inclusions of mutant C-terminal TDP-43 colocalized with either WT or mutant UBQLN2 in co-transfected Neuro2a cells [12]. UBQLN2 directly interacts with the C-terminal fragments of TDP-43 [4]. Moreover, results from our laboratory suggested that the overexpression of either hUBQLN2^WT^ or mutated hUBQLN2^P497H^ in Neuro2A cells can promote the formation of TDP-43 cytoplasmic inclusions [49]. Yet, the mutated form of UBQLN2 induced a stronger cytoplasmic mis-localization of TDP-43 as compared to the WT form. Those results were consistent with other studies that reported the high affinity of TDP-43 and UBQLN2 to form inclusions in the cytosol [4, 12, 16, 27, 34]. The formation of these inclusions has also been reported to be dose dependent. Cells with low expression of WT or mutant UBQLN2 did not exhibit cytoplasmic inclusions of UBQLN2 in TDP-43 aggregates [12, 49].

**Fig. 3:**
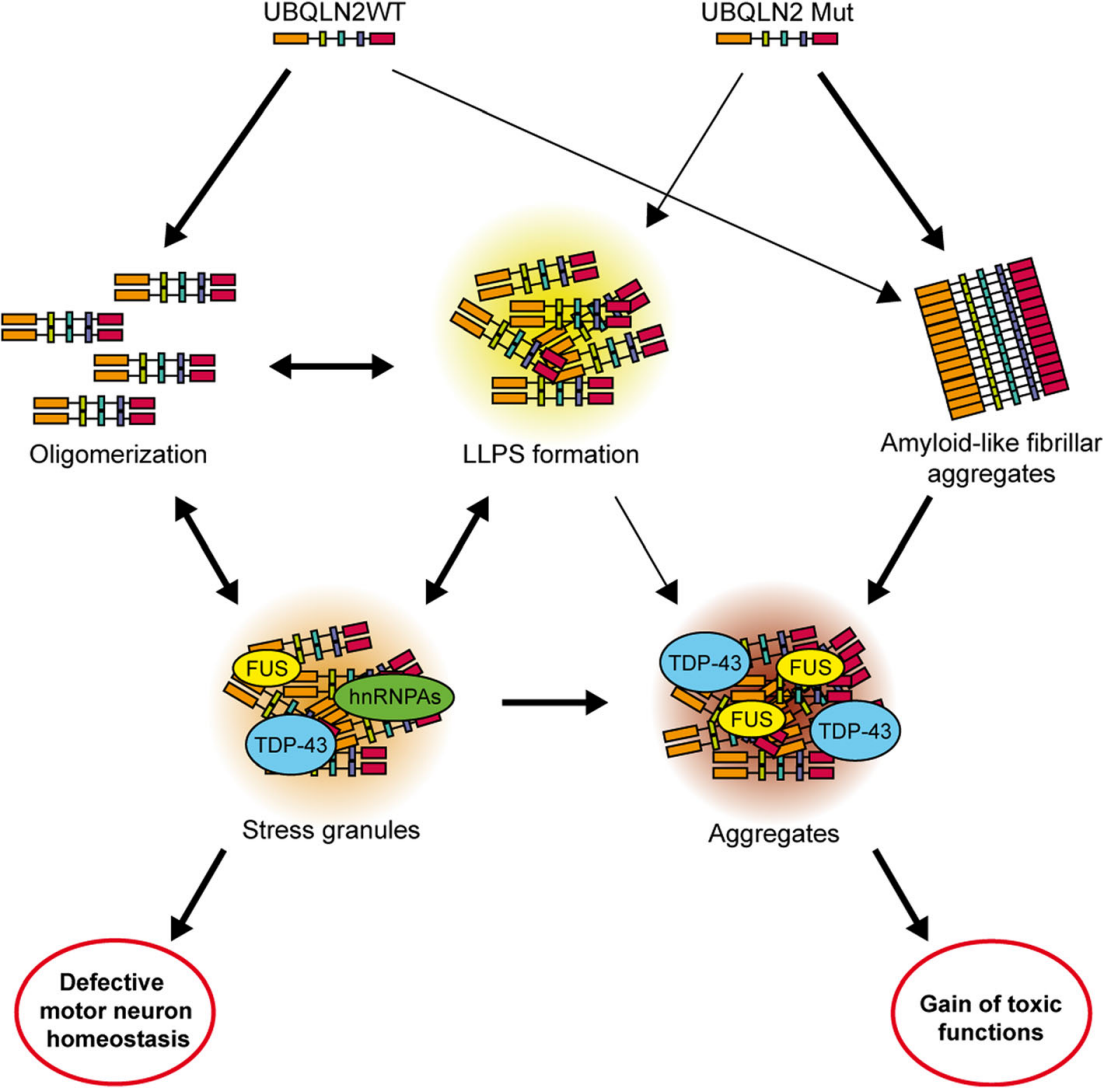
The integrative model for WT and mutant UBQLN2 pathology in ALS. WT UBQLN2 is prone to form oligomers with itself and associates with ubiquitinated substrates, chaperones and other molecules. WT UBQLN2 oligomers can assemble into membraneless organelles and form liquid-liquid phase separation droplets. The mechanism is UBQLN2 levels-dependant. Ubiquitin-binding can prevent this self-assembly and oligomerization of UBQLN2 and an increase of ubiquitin can remove the UBQLN2 LLPS formation. LLPS formations as well as oligomers of UBQLN2 can also become stress granules (SGs). The persistence of SGs can become aggregates or can lead to defect in RNA processing, leading to motor neurons death. Mutant UBQLN2 forms amyloid-like fibrillar aggregates, even in the absence of ubiquitin and chaperones. UBQLN2 mutation increase its insolubility and ubiquitin-binding. A reduced HSP70 binding and autophagy degradation can lead to the formation of aggregates. Aggregation is suspected to be driven by the UBA domain, while the UBL domain seems to inhibit aggregations. UBQLN2WT: Ubiquilin-2 wild-type; UBQLN2 Mut: Ubiquilin-2 mutants; LLPS: Liquid-liquid phase separation; FUS: Fused in sarcoma RNA-binding protein; TDP-43: TAR DNA-binding protein-encoding TDP-43; hnRNPAs: Heterogeneous nuclear ribonucleoprotein A

Strategies to increase ubiquitination have been proposed to boost UPS function [8]. In this regard, our group hypothesised that mutated UBQLN2 may sequester ubiquitin proteins, thereby reducing the degradation of mutated TDP-43 by the UPS and exacerbating the formation of cytoplasmic aggregates [50]. The co-expression, in Neuro2A cells, of hUBQLN2^P497H^ and hTDP-43^G348C^ vectors led to the accumulation of cytoplasmic TDP-43. However, ubiquitin overexpression reduced the cytoplasmic aggregation of TDP-43 [50]. A chymotrypsin-like assay was completed to measure the proteasome efficacy in hUBQLN2^P497H^ and hTDP-43^G348C^ transfected cells. This assay revealed a significant reduction in proteasome efficacy in all transfected cells as compared to control cells. The presence of up-regulated ubiquitin levels corrected the proteasome dysfunction in cells transfected with hUBQLN2^P497H^ and in cells co-transfected with both hUBQLN2^P497H^ and hTDP-43^G348C^. These results suggested that the proteasome impairment caused by hUBQLN2^P497H^ up-regulation may participate to cytoplasmic TDP-43 accumulation. In summary, UBQLN2 overexpression or mutation exacerbated TDP-43 pathology in vitro (Fig. 3). A reduction of UBQLN2 expression or an up-regulation of ubiquitin may constitute potential therapeutic avenues for ALS pathology.

### UBQLN2 in neuroinflammation

The implication of UBQLN2 in inflammation has emerged in the last two to 3 years. The overexpression of hUBQLN2^WT^ and hUBQLN2^P497H^ in Neuro2A cells was found to increase the phosphorylation of p38 MAPK and the nuclear levels of the phosphorylated form of the Nuclear Factor kappa-B (NF-κB), a potent inflammatory mediator [49]. However, it remains unclear if the activation of NF-κB is directly caused by the accumulation of UBQLN2 or comes from the UBQLN2-drived mislocalization of TDP-43 or other mechanisms [58]. Nevertheless, the down-regulation of UBQLN2 with siRNA reversed the increased NF-κB activation. NF-κB is a transcription factor involved in inflammation and probably has a noxious role in ALS and other neurological diseases. Indeed, the RNA levels of NF-κB as well as the levels of TDP-43 were abnormally up-regulated in the spinal cord of ALS patients [58]. TDP-43 was found to interact with p65 NF-κB in spinal cord of ALS but not in control. NF-κB inhibition by withaferin A treatment attenuated disease phenotype in mice expressing mutant TDP-43 and it increased survival in mutant SOD1 mice [14, 15, 48, 58]. Mice harboring UBQLN2^P497H^;TDP-43^G348C^ mutations also exhibited increased levels of NF-κB in spinal cord as compared to TDP-43^G348C^ mice [50]. The double transgenic mice also expressed higher levels of pro-inflammatory cytokines CCL1, CCL5 and CXCL1 and higher stages of activated microglia in the spinal cord as compared to single TDP-43 transgenic mice. Noteworthy, the UBQLN2^P497H^ transgene was only expressed in neurons. The increased inflammation markers could suggest that microglia were hyperactivated in reaction to UBQLN2/TDP-43 neuronal pathology. These results also suggested that UBQLN2 pathology may favorize neuroinflammation and the reduction of UBQLN2 cytoplasmic accumulation may be a potential treatment approach to reduce glial cells activation observed in ALS.

### Stress granules

Stress granules (SGs) are cytoplasmic inclusions of proteins and maintenance mRNAs with the aim of promoting stress response proteins translation in stressful situations, such as oxidative stress, osmotic stress, infection, ER stress and inhibition of the proteasome [3]. Under stress conditions, rather than engaging with the protein degradation system via its UBA and UBL domains, UBQLN2 associates with SGs components through its STI1-like region. UBQLN2 influences the early processes of molecular complex dynamics in phase separation that drives SGs formation. A group recently suggested that UBQLN2 modulates the state of the components to be recruited to SGs rather than regulating the levels of core SGs components [2]. One of the main components identified as UBQLN2-interacting SGs were heterogeneous nuclear ribonucleoproteins A (hnRNPAs) [2, 19]. UBQLN2 has been shown to colocalize with SGs in vitro under different cellular stressor. Indeed, UBQLN2 undergoes liquid-liquid phase separation (LLPS) which correlates with UBQLN2 ability to form stress-induced puncta inside cells [9]. LLPS consist of phase-separation of proteins composed of low-complexity or prion-like domains into membrane-less and spherical compartments. Persistent stress, mutations and aging are suspected to induce liquid-solid phase separation (LSPS) and might become irreversible aggregation, such as amyloid-like aggregates [52, 57]. Also, UBQLN2 puncta formation and stress granules (SGs) colocalization are highly sensitive to levels of UBQLN2 expression.

Stress granules contain various proteins, including fALS related proteins, TDP-43, FUS, hnRNPA1 and 2. A disturbance in stress granules homeostasis can lead to disruption of RNA processing [37] (Fig. 3). It has been recently reported that overexpression of mutated UBQLN2 (P497H and P506T) in HeLa cells directly impaired the binding of UBQLN2 to FUS which resulted in a loss of the ability of UBQLN2 to regulate FUS-RNA complexes and SGs formation [2]. Mutations in FUS and hnRNPA1 have been reported to increase their propensity to accumulate in SGs, disrupting SGs primary function to act as a stress response modulator [30, 36, 47]. Other studies reported that UBQLN2 upregulation can cause a cellular stress leading to MAPK activation, which have been recently shown to have a critical role in TDP-43 accumulation in stress granules following a chronic stress [40, 49].

Ubiquitin is also a common component of SGs [12, 33]. The specific binding between ubiquitin and UBQNL2 could prevent the self-assembly and oligomerization of UBQLN2 [9]. Spectrophotometric assays of ubiquitin and UBQLN2 at different molar ratios, revealed that LLPS formation decreased with the enhancement of ubiquitin. Those results might provide an insight by which UBQLN2 could traffic ubiquitinated substrates from SGs to the proteasome (Fig. 3) [9]. Another group reported that the UBA domain of UBQLN2 drove amyloid-like aggregation of UBQLN2 in vitro and without the help of ubiquitin or chaperones [56]. The UBA domain responsible for binding ubiquitinated substrates was also implicated in the promotion of aggregation because of the propensity of UBQLN2 to self-assemble and then aggregate. That process was modulated by ubiquitin binding to UBQLN2. Indeed, the behavior of the ubiquitin-binding-deficient form UBQLN2^L619A^ in neurons could prevent UBQLN2 self-assembly and could inhibit LLPS of UBQLN2 [56]. Also, expression of UBQLN2^L619A^ induced a 50% increase in neuronal death compared to WT. Collectively, those results suggest that the UBA domain promotes self-assembly of the protein leading to an increase in UBQLN2 aggregation with toxic consequences for neurons.

The LLPS capacity of a protein is based on its multivalent interactions in “sticker” regions which are separated by “spacers” [22]. Recently, a group exposed that ALS-linked mutations, depending of amino acid and sequence position, differently affect the implication of UBQLN2 in LLPS [10]. PXX domain mutations favorize UBQLN2 oligomerization and LLPS formation, creating reversible aggregates which can be cleared by binding to ubiquitin. Mutations to hydrophobic amino acid (P497L, P506A, T487I) increased the interaction between stickers and increased oligomerization of UBQLN2. Inversely, mutations to hydrophile residues (A488T, P500S, P506S, P509S, P525S) have small impact on oligomerization [10]. Increasing the hydrophobicity within “stickers”, but not “spacers”, favorized the oligomerization tendency of UBQLN2 [67]. The sequence position also greatly impacts UBQLN2 capacity for oligomerization. Oligomerization was high with mutation in 497 position and appeared to be little with mutation 500, 509 or 525 positions [10].

## ALS animal models of Ubiquilin-2

Animal models are essential to study in vivo pathological mechanisms as well as to test experimental therapeutic avenues, and numerous animal models were generated for ALS research (recently reviewed in [51]). Several models expressing UBQLN2 mutations have been reported in the past years (Table 2). The first transgenic mouse model was expressing hUBQLN2^P497H^ under the control of the hUBQLN2 promoter [21]. The animals developed cognitive deficits, a dendritic spinopathy as well as UBQLN2 inclusions in the hippocampus. However, no TDP-43 pathology nor loss of motor neurons were reported in this study. This may be explained by the low level of transgene expression. Indeed, in this model, the levels of hUBQLN2^P497H^ was comparable to the levels of endogenous UBQLN2^WT^. Another group used a transgenic hUBQLN2^P497H^ rat model with TRE-doxycycline system [65]. Their model displayed cognitive deficits associated with UBQLN2 aggregates in hippocampus and evidence of neuronal death in cortex at the age of 130 days. Using knocked-in technique, hUBQLN2^P506T^ mice were generated and showed cognitive impairment but no motor deficits [24]. The knocked-in approach resulted in low levels of UBQLN2, which may explain the results.

**Table 2:** UBQLN2 animal models

Species	Mutations	Age at onset (days)	Motor neuron loss	Cognitive deficits	Neuropathological particularities	Ref.
Mice	P497H	30	N	Y	Dendritic spinopathy. Hippocampal NCI (no TDP-43 but with proteasome component, VCP and OPTN)	[21]
Rats	KO P497H	130	N	Y	Hippocampal and cortical neuronal loss, UBQLN2/P62 NCI (no TDP-43), KO rats exhibit no phenotype	[65]
Mice	WT P497H P497S P506T	90	N	Y	UBQLN2 AAV expression, hippocampal and cortical NCI (with UBQLN2, TDP-43, p62, ubiquitin and OPTN), motor phenotype	[5]
Mice	WT P497S P506T	90	Y	Y	Hippocampal and MN NCI (with UBQLN2, TDP-43 and Ubiquitin), motor phenotype, muscle atrophy, NMJ loss, axonal degeneration, gliosis some MN loss and axonal degeneration in WT	[34]
Mice	mP520T (KI)	270	N	Y	Hippocampal, cortical and brainstem NCI (with UBQLN2 and p62) no motor phenotype	[24]
Rats	WT P497H	40	N	Y	Similar phenotype in WT and P497H hippocampal and cortical neuronal loss, NCI (UBQLN2, p62, ubiquitin and RPT1), no motor phenotype	[25]
DM	WT P497H P525S P4X	28 7 14 0	Y	N	NMJ loss in P497H, eye degeneration in mutant, NCI (UBQLN2, ubiquitin and p62) motor phenotype	[31]
Rats	P497H	90	Y	N	Muscle atrophy, axonal degeneration, NMJ loss, MN NCI (UBQLN2, p62, p-TDP-43), motor phenotype no phenotype when expressed in astrocytes	[7]
DM	KO	3	N	N	NCI (UBQLN2, TDP-43 and ubiquitin) motor phenotype	[27]
Mice	UB^P497H^/TDP-43^G348C^	150	Y	Y	Hippocampal and MN NCI (UBQLN2, TDP-43, pTDP-43, ubiquitin and p62), muscle atrophy, axonal degeneration, gliosis, motor phenotype	[50]

*WT* wild-type, *Y* yes, *N* no, *n/a* not applicable, *HTT* huntington disease protein, *NCI* neuronal cytoplasmic inclusions, *MN* motor neurons, *NMJ* neuromuscular junction, *KI* knock-in, *DM Drosophila melanogaster*

Transgenic mice bearing Thy1.2-driven hUBQLN2^P497S^ or hUBQLN2^P506T^ mutations exhibited motor neurons loss and cognitive impairments [34]. The levels of mutated UBQLN2 expression in those mice were 70–80% of the endogenous mouse UBQLN2 but the phenotypes were not consistent within all the animal cohorts. Indeed, only 10% of the transgenic hUBQLN2^P497S^ mice and 40% of hUBQLN2^P506T^ mice developed hindlimb paralysis. Interestingly, TDP-43 mislocalization could be noted in neurons. Transgenic mice overexpressing hUBQLN2^WT^ also showed loss of upper motor neurons as well as hippocampal neurons. Nevertheless, the effects were less important than those observed in the mutant transgenic mice. Knockout models were also generated, but phenotypes were highly divergent between species (Table 2). While knockout rats did not exhibit any phenotype [65], UBQLN2 knockout in *Drosophila melanogaster* (DM) created a severe motor phenotype with TDP-43/Ubiquitin/UBQLN2 aggregation [27]. The cause for this discrepancy is unknown, but could potentially be explained by the fact that DM carry only one human UBQLN2 orthologue (dUbqn), as compared to rats and human, which possesses four members of the UBQLN family [35]. Consequently, other members of the UBQLN family could possibly compensate for UBQLN2 functions in rats.

Lastly, our team generated the first double transgenic mouse model harboring both hUBQLN2^P497H^ and hTDP-43^G348C^ mutations using a NFH promoter with low levels of expression [50]. The simple transgenic mice hUBQLN2^P497^animal models are summarized in Table 2.

## Conclusion and future directions

Collectively, these multiple reports suggest that UBQLN2 is acting on various ALS pathological mechanisms and is not only causing an UPS dysfunction as previously recognized. It is now documented that UBQLN2 is acting on stress granules formation, on TDP-43 mislocalization into cytoplasmic inclusions, on cellular clearance pathways and neuroinflammation. Thus far, the pathological role of mutated UBQLN2 is more defined, but many questions remain on the exact role of the WT UBQLN2 in sporadic cases of ALS. Nevertheless, the tight control in the levels of UBQLN2 appears to be a primary factor for its role in the clearance pathway and stress granules formation. A small disturbance in its levels could promote oligomerization and trigger dysfunction in LLPS formation, inducing aggregates formation and causing toxicity in neurons. To our knowledge, the levels of UBQLN2 in tissues from sporadic ALS cases without UBQLN2 mutation have never been evaluated nor neuropathological studied and this question should be addressed. Indeed, the WT UBQLN2 form also appears to have a toxic role when overexpressed [12, 49]. It would be of interest to study the relationship between levels of UBQLN2 and the proteasome dysfunction in sporadic ALS. The down regulation of UBQLN2 using antisense oligonucleotide (ASO) or other gene therapy approaches could be a potential strategy for cases with genetic UBQLN2 mutations. This could have an impact on the efficacy of the proteasomal and autophagy clearance pathways to reduce toxic protein accumulation in motor neurons. While, UBQLN2 may provide a promising target for the development of new therapeutics, more work is needed to analyze the potential impact of reducing UBQLN2 levels with therapies in familial and sporadic diseases.

## Data Availability

Not applicable.

## Abbreviations

ALS: Amyotrophic lateral sclerosis
APG9A: Autophagy-related protein 9a
ATG16: Autophagy-related 16
C9ORF72: Chromosome 9 open reading frame 72
CCL1: C-c motif chemokine ligand 1
CCL5: C-c motif chemokine ligand 5
CXCL1: C-x-c motif chemokine ligand 1
ER: Endoplasmic reticulum
ERAD: Endoplasmic-reticulum-associated protein degradation
fALS: Familial amyotrophic lateral sclerosis
FTD: Frontotemporal dementia
FUS: Fused in Sarcoma
HMWCs: High-molecular-weight complexes
hnRNPA1: Heterogeneous nuclear ribonucleoprotein A1
HSP70: Heat shock protein 70
LLPS: Liquid-liquid phase separation
LSPS: Liquid-solid phase separation
MAPK: Mitogen-activated protein kinases
NF-κB: Nuclear Factor kappa-B
OPTN: Optineurin
RNA: Ribonucleic acid
sALS: Sporadic amyotrophic lateral sclerosis
SGs: Stress granules
SH3: Src homologue 3 protein
SOD1: Super-oxide dismutase 1
STI-I: Stress induced protein 1-like
TDP-43: TAR DNA-binding protein 43
UBA: Ubiquitin-associated domain
UBL: Ubiquitin-like domain
UBQLN2: Ubiquilin-2
Ubxd8: Ubiquitin regulatory X domain-containing protein 8 protein
UPS: Ubiquitin-proteasome system
WT: Wild-type
